# Using graphene networks to build bioinspired self-monitoring ceramics

**DOI:** 10.1038/ncomms14425

**Published:** 2017-02-09

**Authors:** Olivier T. Picot, Victoria G. Rocha, Claudio Ferraro, Na Ni, Eleonora D'Elia, Sylvain Meille, Jerome Chevalier, Theo Saunders, Ton Peijs, Mike J. Reece, Eduardo Saiz

**Affiliations:** 1 School of Engineering and Materials Science, Queen Mary University of London, Mile End Road, London E1 4NS, UK; 2 Department of Materials, Centre for Advanced Structural Ceramics, Imperial College London, London SW7 2AZ, UK; 3 Université de Lyon, INSA Lyon, MATEIS CNRS UMR5510, F-69621 Villeurbanne, France; 4 Nanoforce Technology Limited, Mile End Road, London E14NS, UK

## Abstract

The properties of graphene open new opportunities for the fabrication of composites exhibiting unique structural and functional capabilities. However, to achieve this goal we should build materials with carefully designed architectures. Here, we describe the fabrication of ceramic-graphene composites by combining graphene foams with pre-ceramic polymers and spark plasma sintering. The result is a material containing an interconnected, microscopic network of very thin (20–30 nm), electrically conductive, carbon interfaces. This network generates electrical conductivities up to two orders of magnitude higher than those of other ceramics with similar graphene or carbon nanotube contents and can be used to monitor ‘*in situ*' structural integrity. In addition, it directs crack propagation, promoting stable crack growth and increasing the fracture resistance by an order of magnitude. These results demonstrate that the rational integration of nanomaterials could be a fruitful path towards building composites combining unique mechanical and functional performances.

## Abstract (web summary)

Micro- and nanostructures found in nature can be adopted to new uses and materials in engineered composites. Here authors demonstrate large enhancements in toughness and electrical conductivity in a ceramic upon addition of graphene at low (1 volume %) levels.

The development of new technologies in key areas from construction to transportation to energy generation increasingly demands new structural materials with improved performance. These materials will not only have to be lighter, stronger and tougher but also play additional functional roles, including sensing external stimuli, self-monitoring their structural health, conducting electricity and storing energy. It is becoming evident that monolithic materials cannot meet these stringent demands. Consequently, researchers are looking at the development of new composites as a solution to meet the challenge. As the work progresses, it is becoming increasingly clear that, to reach the desired performance, the structure of these composites will have to be carefully designed at multiple length scales from the atomic to the macro level. For example, an increasing body of work has been devoted to the integration of carbon nanostructures such as carbon nanotubes (CNTs) or graphene, in ceramic or polymer matrices. While there have been some promising results, in terms of mechanical response, the outcome has been inconclusive at best^1^,^2^,^3^. The unique intrinsic mechanical properties reported for these nanostructures are often based on modelling or experiments performed at the nanoscale. However, translation to materials at practical dimensions is extremely complicated and involves structural design and processing. For example, it has been recently shown how defects can lead to significant variations in the strength of graphene^4^. Furthermore, in many applications strength is not the limiting practical property, fracture resistance is, which opens the fundamental question: how to effectively use nanoscale reinforcements to promote fracture resistance when in composites toughness is often generated through extrinsic mechanisms that act at much larger length scales (from the microscopic and upwards). Moreover, improved mechanical performance alone may not be enough to warrant the use of nanofillers, which should also provide functional capabilities. A possible solution to this problem is to move away from the traditional approach that sees all these carbon nanomaterials as ‘reinforcements' dispersed in a matrix and instead use their unique dimensionality to engineer complex microstructures that can deliver the desired properties and functionalities.

Graphene-ceramic composites are being investigated for many different applications, from protective coatings, to energy storage or medicine^5^,^6^,^7^. Diverse techniques (from colloidal processing to chemical vapour deposition) have been used to fabricate composites with a homogeneous distribution of graphene nanoplatelets^8^,^9^,^10^. Some degree of toughening has been observed in these materials and crack bridging by the graphene nanoplatelets has been identified as one of the main mechanisms^11^,^12^,^13^. However, because of the relatively small size of the platelets the degree of bridging is limited. To trigger additional toughening it is necessary to manipulate the platelet distribution but this has proven challenging. There are reports of the use of layered arrangements prepared by sequential powder stacking to improve mechanical and electrical performance. It is difficult to refine the microstructures using these approaches and the thickness of the graphene containing layers is usually above a micron with ceramic layers >50 μm thick^14^,^15^.

Material scientists are increasingly looking at the design of natural structural mineralized composites such as bone or nacre in search of new design concepts^16^. These natural materials develop unique mechanical response from very simple components. They usually exhibit complex anisotropic architectures with layered, columnar or fibrous motifs^17^. Furthermore, to a large extent their properties depend on the careful engineering of interfaces at the chemical and structural levels. These design concepts have also been employed in synthetic composites and, in particular, weak interfaces are often used in ceramic-based materials as a way to promote toughness through mechanisms such a crack deflection or fibre pull-out^18^. Here graphene opens new opportunities as its two-dimensional (2D) structure is very well adapted to interfacial engineering. Carbon has been used before to create weak interfaces, for example, in layered ceramic materials^15^,^19^,^20^. However, in these systems the ceramic layers are usually hundreds of microns thick and the interfaces are also in the micron range and relatively flat. Nature also uses interfacial roughness to promote friction during crack propagation and enhance fracture resistance. This strategy has been more difficult to replicate synthetically but, for example, Mirkhalaf *et al*.^21^ increased the toughness of glass by laser engraving wavy internal interfaces. However, the waviness and layer thickness were in the hundreds of micrometers range and the procedure significantly reduced the strength of the material. One of the critical features of some natural systems that has been very difficult to replicate synthetically is the presence of very thin (few nanometres) soft interfaces separating hard, mineral layers^16^. In general, and with few exceptions, the architectural motives of most synthetic structures are still orders of magnitude larger than in their natural counterparts^22^,^23^.

To address these issues, in this study we take advantage of the 2D nature of chemically modified graphene (CMG) to engineer a fine network of internal interfaces in a glass ceramic matrix. We use networks of CMG as the starting point to create composites with layered architectures and an interconnected carbon grid. The use of graphene allows the engineering of very thin (<20 nm) nano-rough, interfaces. Despite the fact that the matrix is brittle and constitutes ∼99 vol% of the material, these interfaces promote stable crack growth and a fracture resistance up to an order of magnitude (in terms of energy) higher than that of the glass ceramic. They also provide a highly electrically conductive network (conductivity>500 S m^−1^) that can be used to sense the formation and progress of damage. This combination of self-monitoring and high-fracture resistance can be used to develop intelligent materials able to avoid catastrophic failure in service and illustrate a different approach for the integration of graphene in ceramic and polymer-based composites as opposed to the traditional ‘reinforcement' strategy.

## Results

### Building graphene/ceramic composites

**Figure 1:**
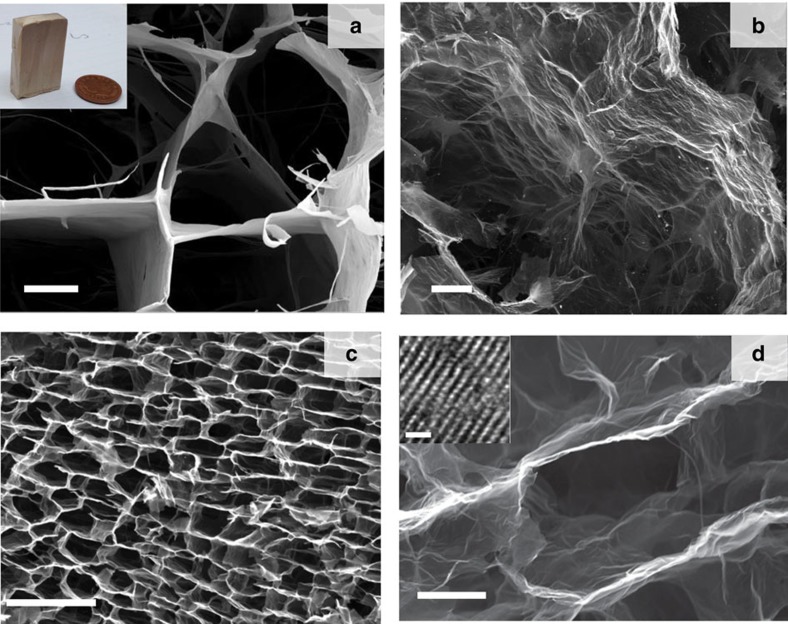
CMG networks. Scanning electron micrographs of the CMG network (taken using secondary electrons) (**a**) before and (**b**–**d**) after thermal reduction. The inset in **a** shows the sample size. The images **a** and **b** were taken in the plane perpendicular to the direction of ice growth during freeze casting, while **c** and **d** were taken in the parallel plane. Comparison of **a** and **b** shows that thermal reduction results in wrinkling and roughening of the network walls, while the comparison of **a** and **d** illustrates the shrinkage or the cells. The network shows large scale ordering (**c**) retaining very thin walls after reduction (**d**). The inset in **d** shows a high-resolution transmission electron micrograph of the cross section of one of the network walls showing the stacking of the carbon layers. Scale bar, 10 μm (**a**) and (**b**); 100 μm (**c**); 5 μm (**d**); 10 Å (**d**) inset.

**Figure 2:**
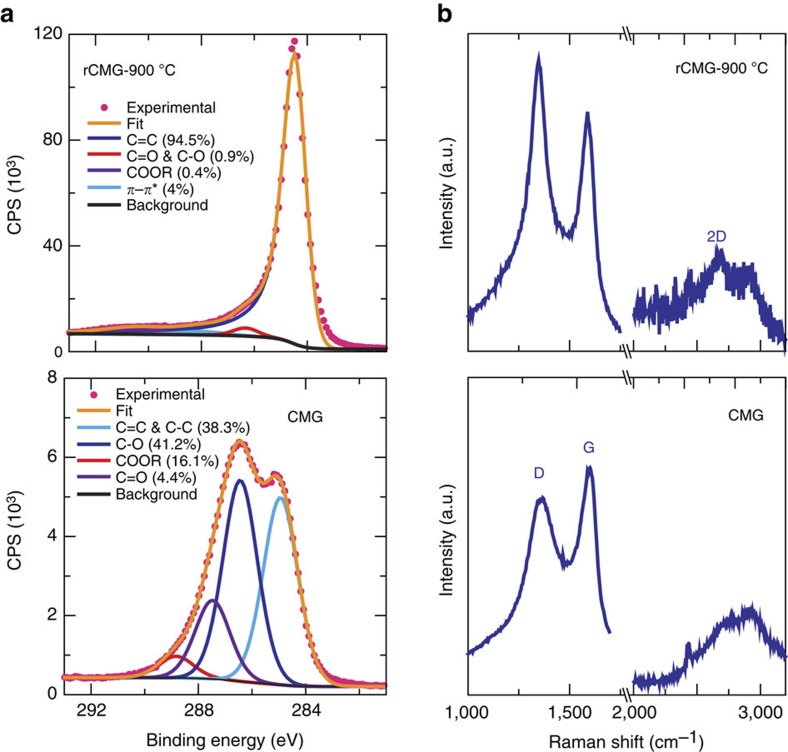
Reduction of the CMG networks. (**a**) XPS analysis of the networks before and after reduction. The XPS C1 signals were fitted to five components: C=C and C–C, C–O, C=O and COOR for the as-prepared ice template CMG network and C=C, C=O and C–O, and COOR and the *π*–*π* shake up for the reduced CMG network. The rCMG atomic composition is C1s (79.1%), N1s (0.5%), O1s(12.0%), Si2p (7.6%), Mn2p (0.6%), Na1s (0.3%). Silicon and Nitrogen are attributed to the vacuum grease used during the freeze casting process while the small fraction of Manganese could be a residue from the synthesis route. (**b**) Corresponding Raman analysis. Thermal reduction results in sharper D and G peaks and, the appearance of the 2D peak at 2,700 cm^−1^ which is characteristic of graphene.

The first step of our process is the fabrication of CMG networks using freeze casting^24^,^25^. Graphene oxide (GO) suspensions in water were prepared through the chemical exfoliation of large flake graphite using a modified Tours' method^26^. Diverse organic additives (PVA:sucrose in a 1:1 fixed weight ratio and with a total content varying between 0.2 and 2 wt.%) were added to the concentrated GO suspensions (2–20 mg ml^−1^) to bond the GO flakes after the freeze-casting process. The suspensions were frozen directionally to form 3D porous networks with macroscopic dimensions and a characteristic anisotropic layered architecture templated by the ice. The internal structure of the network consists of long microscopic channels oriented along the ice growth direction Their diameter is of the order of ≈20–30 μm and they are separated by thin (20–30 nm) walls formed by the confined rearrangement of GO flakes between growing ice crystals during freezing (Fig. 1; Supplementary Fig. 1). Subsequently, the networks were freeze dried and heat treated at 900 °C in a reducing atmosphere to form reduced CMG (rCMG). After the reduction, the network walls are extensively wrinkled (Fig. 1). There is a 20% volume shrinkage after the treatment. The thermal reduction process is complex and involves the removal of oxygenated functional groups (epoxy and hydroxyl functional groups present on the basal planes and carbonyl, carboxyl groups located at the sheet edges) in the form of gaseous species through the porous network, defect formation, lattice contraction, folding and unfolding of the layers and layer stacking. All contribute to some extent to an effective restoration of the *sp*^2^ carbon network. This was confirmed by X-ray photoemission spectroscopy (XPS) and Raman analysis (Fig. 2 and Supplementary Fig. 2). XPS characterization indicates that the C/O ratio increased from 1 to 12 after thermal reduction. Interestingly, the changes were not as clearly reflected in the Raman spectra presumably due to the low annealing temperature. Here the main features are the so-called D and G peaks, which lie around 1,360 and 1,560 cm^−1^ respectively. Thermal reduction results in sharper D and G peaks and, more importantly, the appearance of the 2D peak at 2,700 cm^−1^, which is characteristic of graphene^27^. The combination of freeze casting with the use of high-purity CMG flakes with large (lateral size >10 μm, Supplementary Fig. 3) results in strong networks that can withstand very large deformations^24^,^25^.

**Figure 3:**
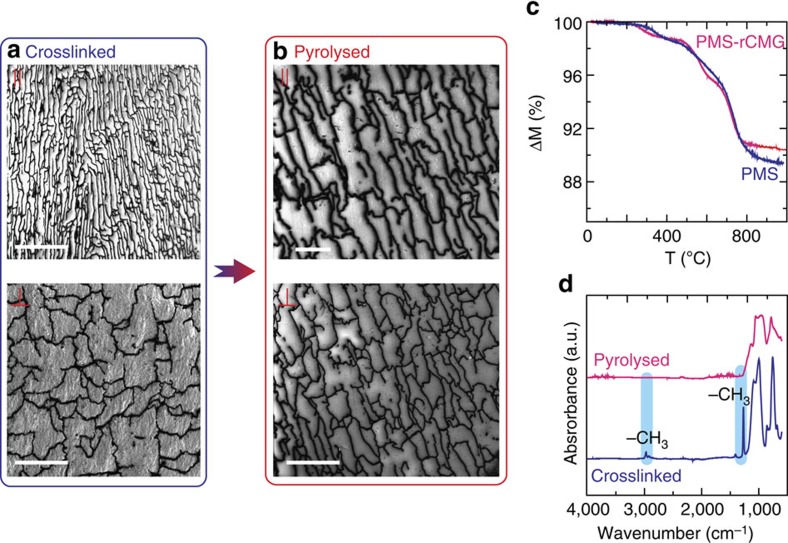
Characterization of the composites after infiltration and pyrolysis. (**a**) SEM micrographs (taken using secondary electrons) showing that the graphene network has been fully infiltrated and maintains its interconnected structure after crosslinking (parallel to the ice growth direction, ‖, and perpendicular, ⊥). (**b**) A dense composite is obtained after pyrolysis. (**c**) The thermogravimetric analysis indicates that the polymer pyrolysis is not affected by its confinement in the graphene network. Weight losses occur at the same temperature in the pure polymer and in the composite and cease around 800 °C. (**d**) FTIR analyses before (crosslinked) and after pyrolysis shows that the thermal treatment has been effective in the conversion to an inorganic material. The peaks corresponding to the asymmetric stretching (∼2,900 cm^−1^) and deformation (∼1,250 cm^−1^) of the -CH_3_ groups have disappeared. Scale bar, 200 μm (**a**) top; 50 μm (**a**) bottom; 25 μm (**b**) top; 50 μm (**b**) bottom.

These 3D porous networks were infiltrated with polymethyl siloxane, a pre-ceramic polymer which was chosen for its high ceramic yield (90%), and heat treated up to 1,000 °C in N_2_ to convert the siloxane polymer to a silicon oxycarbide glass. The hydrophobic nature of rCMG facilitates complete infiltration^24^. Because of their mechanical properties the networks can maintain their shape and structure during polymer infusion and crosslinking. They shrink with the matrix as the polymer converts to a ceramic during heating. The result is a dense material (Fig. 3). The remaining silane that does not polymerize is removed around 350 °C during pyrolysis and the polymer continues to degrade forming gaseous products such as methane and/or hydrogen up to 700 °C (ref. 28) (Fig. 3c). The presence of graphene did not affect the degradation process and the overall ceramic yield was around 90%. FTIR studies (Fig. 3d) confirmed the organic–inorganic conversion. The graphene network is essential to maintain the structural integrity of the composite during conversion. The polymer alone cracks into a glass powder during pyrolysis due to the stresses that can be generated if there is some inhomogeneity during conversion (and therefore in the associated dimensional changes) and also due to the liberation of gaseous species^29^. In the composite, the network walls are able to hold the material during pyrolysis and maintain integrity (Fig. 3b). A method has been recently proposed to fabricate dense bulk ceramics in which the polymer is used to infiltrate a proprietary scaffold followed by pyrolysis. It has been proposed that the scaffold facilitates gas release during heating to avoid pressure build up^29^. However, it is not clear that the carbon network can facilitate gas release in our system.

### Composite structure and chemistry

The density of the pyrolysed samples is ∼1.9±0.1 g cm^−3^, in good agreement with results reported in the literature for polymer derived Si–O–C (ref. 29). To increase the density of the composite and further restore the *sp*^2^ network of the rCMG, the samples were sintered by Spark Plasma (SPS). During SPS, nano- and micropores are closed by the combined effect of the applied pressure and creep deformation of the matrix^30^. The glass transition temperature of Si–O–C is around 1,350 °C, hence higher temperatures are required to reach full densification. Although the heating rate during SPS is fast (100 °C min^−1^) and the dwell is only 8 min, Si–O–C glasses undergo a series of structural and chemical changes at high temperature such as bond redistribution and phase separation^29^,^31^. Comparison of the material with and without graphene suggests that these changes are not affected by the presence of the network (Supplementary Fig. 4). The optimum sintering temperature is 1,700 °C which resulted in a 20% increase in density. Higher temperatures (1,900 °C) led to a drop in density due to further loss of oxygen from the Si–O–C and the formation of pores. The final content of rCMG in the composite is ∼1 vol.%.

**Figure 4:**
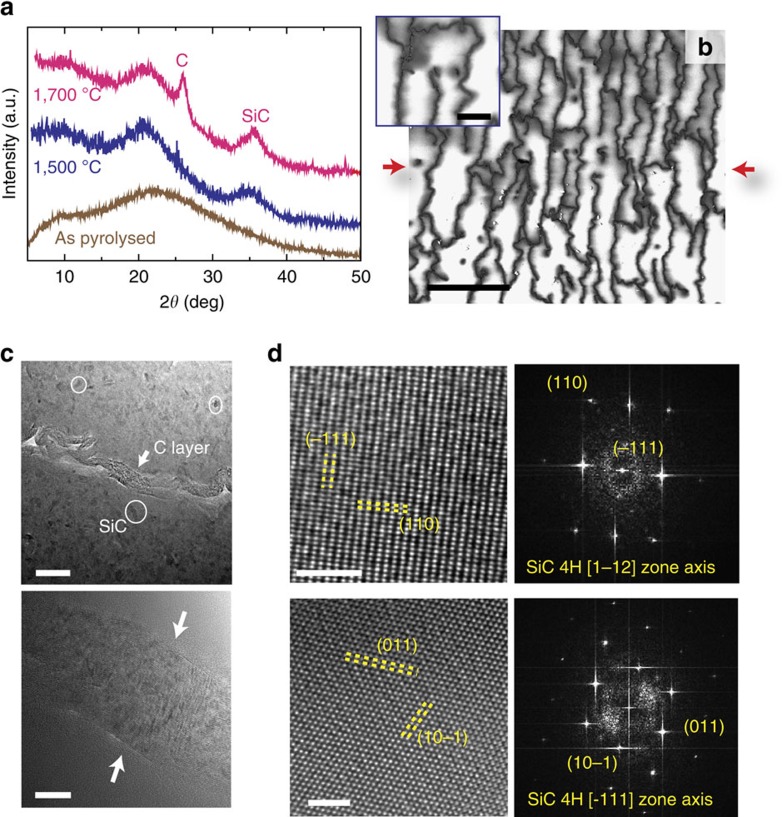
Composites after SPS. (**a**) X-ray diffraction analysis of the sample after SPS showing the crystallization of SiC at 1,700 °C. (**b**) SEM micrograph (plane parallel to the ice front) showing the corrugated walls of the graphene network. The interfaces are rough at the micro to nano scale. The red arrows correspond to the pressure direction during SPS. The images have been taken using secondary electrons with non-coated samples to enhance contrast between the matrix and the interfaces. As a result a grey halo is visible around the carbon interfaces. (**c**)TEM micrographs showing two different carbon interfaces (∼20–30 nm thick) at high magnification. The interfaces (marked with the white arrows) are generated by the rCMG network encapsulated in a glass-ceramic matrix formed by nanocrystals (in the white circles) dispersed in glass. (**d**) High-resolution TEM analysis confirms that the nanocrystals are SiC. Scale bar, 25 μm (**b**); 5 μm (**b**) inset; 50 nm (**c**) top; 10 nm (**c**) bottom; 2 nm (**d**).

After SPS, the sample retains a layered structure in which an interconnected rCMG network is immersed in a glass-ceramic matrix (Fig. 4). SPS at temperatures>1,500 °C induced partial crystallization of SiC (Fig. 4d). Higher sintering temperatures (1,700 °C) lead to the apperarance of an additional X-ray diffraction peak at 26° that can be attributed to the formation of graphitic domains in the matrix as confirmed by Raman spectroscopy. It should be pointed out that characterisation data (X-ray diffraction and Raman) for the composite are dominated by signal from the matrix phase that includes free carbon and therefore it is not possible to clearly comment on the state of the rCMG (Supplementary Fig. 4). However, the Raman spectra of the composite sintered at 1,700 °C when compared with 1,500 °C ones show a sharper 2D peak at 2,700 cm^−1^ and lower *I*_D_/*I*_G_ ratio as a consequence of an increase in the graphitic order of the graphene network and the free carbon in the matrix (Supplementary Fig. 4). After SPS, the network walls remain extensively crumpled and wrinkled (Fig. 4b,c). The crumpling is more evident in the walls perpendicular to the applied pressure. Their thickness ranges between 20 to 30 nm and they are composed of rCMG sheets self-assembled into a regular arrangement induced by the ice growth. There is no apparent reaction or interfacial layer between the ceramic matrix and the rCMG. The Young modulus of the network walls is relatively low (10 GPa or the order of graphene paper)^25^. This could be expected as the walls are formed by the entanglement of graphene flakes held together by van der Walls type of forces. In addition, there is a very large difference in the thickness of the graphene and ceramic layers (the ratio is below 1/100). Therefore, it could be expected that the network does not constrain the dimensional changes associated to pyrolysis, sintering and shrinkage during cooling after SPS and that the thermal stresses in the ceramic remain low.

### Mechanical performance

The carbon network modifies the fracture behaviour of the matrix. The glass-ceramic exhibits the expected brittle behaviour with catastrophic failure and low initiation toughness (*K*_IC_∼0.80±0.03 MPam^−1/2^). The corresponding critical strain energy release rate is 6.3±2 J m^−2^, calculated as:


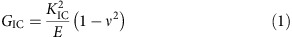


Where *E* and *ν* are the Young modulus and Poisson ratio of the material (100 GPa and 0.11, respectively)^32^. The work of fracture measured from the area under the stress strain curve is 28.7±5 J m^−2^. However, this is just an upper limit as fracture is unstable in this material with the value calculated from equation (1) being a closer estimate.

**Figure 5:**
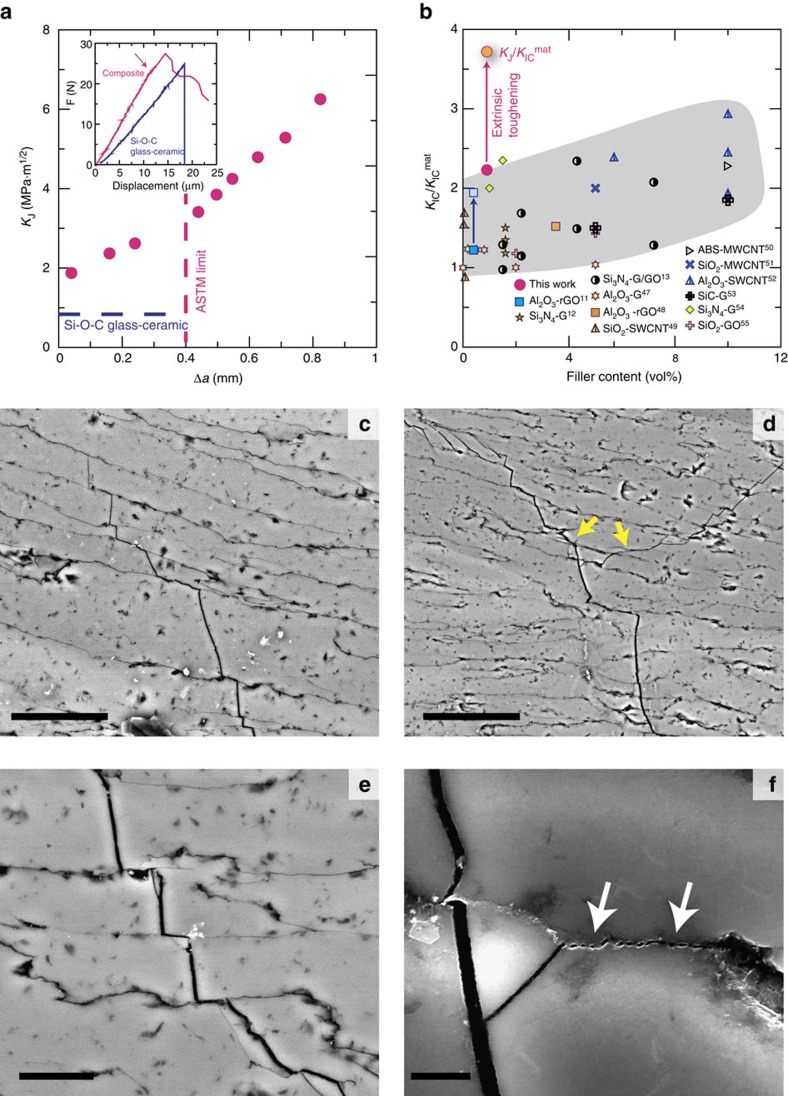
Mechanical response. (**a**) While the glass-ceramic is brittle, the composite exhibits stable crack-propagation (insert shows a typical force-displacement curve with the arrow indicating crack initiation, differences in the load-displacement curve slope are probably due to differences in sample and notch sizes rather than to a differences in stiffness) with rising resistance curve (*R*-curve). The maximum toughness (in terms of *K*_J_) is of the order of 3–3.5 MPa m^1/2^ (within the ASTM limit) (**b**) When comparing the fracture resistance to those of other ceramics reinforced with carbon nanostructures it is possible to see that the interconnected network offers very efficient toughening at extremely low carbon contents (*K*_IC_ is the toughness of the composite and *K*_IC_^mat^ that of the matrix material). When taking into account extrinsic toughening (as measured in the *R*-curve) the increase (comparing the final resistance, *K*_J_, for the longer cracks with the toughness of the Si–O–C matrix) is several times higher. (**c**–**f**) Several toughening mechanisms could be identified in the scanning electron micrographs taken of the propagating crack. These include crack deflection (**c**–**e**), branching (**d**) and interfacial friction (**f**). These parallel those observed during the fracture of a natural layered material: nacre. All SEM images in this figure where taken using backscattered electrons. Scale bar, 25 μm (**c**); 50 μm (**d**); 10 μm (**e**); 2 μm (**f**) (refs 11, 12, 13, 47, 48, 49, 50, 51, 52, 53, 54, 55).

The initiation toughness in the composite is of the order of 1.7±0.1 MPa m^1/2^, double that than that of the glass-ceramic matrix. More importantly, even though the composite contains only 1 vol.% of carbon it is possible to achieve stable crack propagation. As a result, it exhibits a rising *R*-curve behaviour with a steady rise of *K*_J_ (Fig. 5a). The maximum toughness values within the limits of the short bridging regime are of the order of 3–3.5 MPa·m^1/2^ (according to ASTM E1820-13), which is ∼3.8 times higher than for the pure glass-ceramic (in terms of *K*), which corresponds to∼14 times higher in terms of energy (∼88 J m^−2^, taking *K*_J_∼3 MPa m^1/2^ and assuming that 1 vol% graphene does not have a substantial effect on the Young modulus and Poisson ratio). As we have stable crack propagation we can calculate the corresponding work of fracture from the stress strain curve, and it is of the order of 46.6±6 J m^−2^ that again is 7 to 8 times higher than the closer estimate for the glass-ceramic.

The CMG has formed a continuous network of weak interfaces that promote microcracking, crack branching and deflection (Fig. 4c–f). These interfaces are thin (approximately 20–30 nm) and rough at the microscopic scale, and friction between the sliding glass layers/blocks adds another source of energy dissipation during crack propagation. Because of the crack deflection along the interfaces the ceramic layers can also provide some degree of bridging in a way akin to what has been observed in SiC or Si_3_N_4_ materials combining intergranular fracture with large aspect ratio grains. The increase in toughness (in terms of K) is comparable to the ones measured in these systems^33^,^34^,^35^. The role of interfacial roughness has been highlighted by the work of Mirkhalaf *et al*.^21^ where they are able to double the fracture toughness (in terms of energy) by engraving interlocking interfaces in a glass. However, this was done at the expense of the strength which was reduced to 10% of its original value. Here we have reduced the characteristic dimensions of the ceramic layers down to 10–20 μm. In addition, by using CMG as a precursor we have kept the thickness of the interfacial layers below 30 nm while the overall carbon content of the material is about 1 vol.%. One of the main challenges found in the development of synthetic nacre-like materials has been to reduce the thickness and overall content of the ‘soft mortar' phase to values comparable to their natural counterparts. The use of an atomically thin flake allows the assembly of such thin interfaces to maintain up to 99 vol.% ceramic in the material and retain a significant amount of strength (63±2 MPa for the composites vs 120±5 MPa for the pure Si–O–C ceramic, with the decrease probably due to the introduction of the interface network).

It is interesting to compare the properties of the system with ceramic laminates that use C or BN to form a thin, relatively weak interface between SiC or Si_3_N_4_ layers^19^,^20^,^36^. In these composites the thicknesses of the ceramic layer and the interfaces are one to two orders of magnitude larger than those of the materials described here. In addition the layers are flat and continuous with lengths of centimetres. The samples are usually tested in bending with the load applied perpendicular to the layers. Increases of the work of fracture between two to three orders of magnitude have been reported in these materials. However, one of the main causes seems to be crack deflection. Cracks form and run along the interfaces in some cases even before the maximum force is reached in the load deflection curve and they can run for distances of up to millimetres in the set-ups used in the papers. The material described here is closer to a brick and mortar structure and crack deflection is much more limited. In addition, the work on layered materials used high-performance technical ceramics where here we have used a brittle glass-ceramic to prove the concept.

In this work, the samples were tested in bending. If we assume the structure close to a brick and mortar, when testing in tension the ratio between the strength of the ceramic bricks and the adhesion strength of the interface dictates the optimum brick aspect ratio to achieve maximum strength while maintaining interfacial crack propagation to maximize toughness^37^. As the thin carbon interface is relatively weak interfacial failure is most likely. A close system to this could be the laser-engraved glass where a significant degree of brick sliding, crack bridging, crack deflection and branching has been observed in tension^21^,^38^. We could expect similar toughening mechanisms acting here under similar loading conditions. The results suggest that in these systems brick interlocking (as given for example by the interfacial roughness) and the properties of the mortar will determine to a large extent the degree of toughening.

### Sensing damage

**Figure 6:**
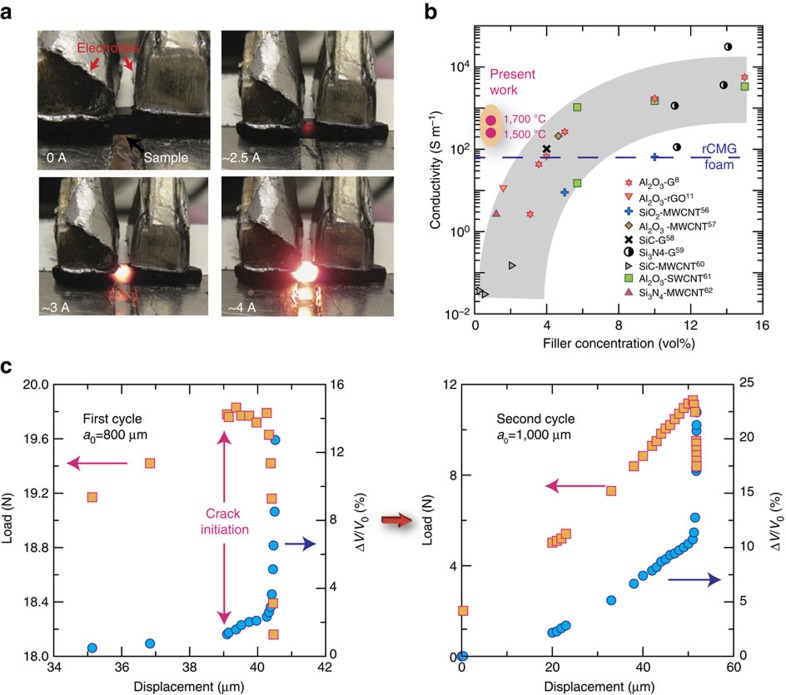
Electrical properties. (**a**) Demonstration of the Joule heating effect of the composite (distance between electrodes ∼0.7 mm). (**b**) Due to its finely interconnected network the electrical conductivities are one to two orders of magnitude higher than for ceramic materials with similar contents of carbon nanofillers (data for composites sintered at two temperatures). (**c**) Load displacement curves taken during the 4-point bending of a notched sample and simultaneous variation of the voltage taken in using a 4-probe set-up (constant current). The partial disruption of the graphene network by crack formation and propagation can be used to monitor the materials structural integrity. In the first cycle, there is a measurable increase in voltage when a cracks starts to form and propagate. The test was stopped after the crack underwent a length increment of ∼200 μm. Changes in voltage can be detected during initiation when the crack is few microns long (voltage baseline is taken for an initial notch length, *a*_*o*_=800 μm). When the load is released, the crack closes and connection in the network is recovered. After the load is re-applied (second cycle) a gradual increase in voltage is seen almost immediately corresponding to crack opening followed by a step increase when the it starts to propagate^8^,^11^,^56^,^57^,^58^,^59^,^60^,^61^,^62^.

The continuous interfaces provide a highly interconnected conductive network that can act as an efficient Joule heater or can be used to monitor the formation of defects and crack propagation (Fig. 6). The composite exhibited anisotropic electrical conductivity as could be expected from its structure. Measurements performed along the ice growth direction gave values one order of magnitude higher than perpendicular to it (500 versus 33 S m^−1^ for samples sintered at 1,700 °C). Despite the formation of SiC and free C in the matrix, our measurements indicate that the electrical conductivity of Si–O–C is at least two to three orders of magnitude below that of the composite. Therefore its contribution to the conductivity is negligible. These conductivities are significantly higher than those of the reduced network alone (up to 60–70 S m^−1^ in the ice growth direction) and depend on the sintering temperature. The electrical conductivity for the composite sintered at 1,500 °C was found to be half of that prepared at 1,700 °C. This is consistent with previous findings that indicate that high temperature treatments in carbon containing atmospheres (SPS or hot pressing) are very efficient in improving the crystallinity of CMG and increasing its electrical conductivity^24^. In all cases the measured conductivities are one to two orders of magnitude larger than those of ceramic-carbon composites with similar nano-carbon contents (Fig. 6). This is due to an approach that allows the formation of an interconnected carbon network in a much more efficient way than through the random dispersion of particles, nanotubes or nanoplatelets. We have used a 4-point probe set up to measure ‘*in situ*' the variation in voltage (constant current) during bending a notched bar of the composite like the one used to calculate the *R*-curve (Fig. 6c). The measurement clearly shows a rise in voltage due to microcracking, before failure (and before the maximum stress has been reached). Since crack propagation is stable, the load can be released before fracture and the voltage is recovered as the network recovers its connectivity. In absolute values, and with the set-up and current (20 mA) used in this test, a voltage increase of 1.5 mV was measured for a crack of 200 μm. In a second cycle, the same phenomena can be observed. However, in this case the voltage rises first before the crack starts growing again as a consequence of the opening of the existing crack that breaks the network. It then reaches a value close to that observed in the first cycle and subsequently there is a sharp increase in voltage corresponding to crack growth followed by failure.

## Discussion

Carbon nanomaterials such as graphene or nanotubes have extraordinary intrinsic properties. They can combine high strength and stiffness with functional properties such as high electrical and thermal conductivity. However, to incorporate them in synthetic composites that take full advantage of their potential, their random dispersion in a matrix will not be sufficient. A high theoretical strength for a pristine material often does not translate into practical structures. In addition, strength is not the only design criteria in the selection of materials, other properties such as toughness are equally or more important, and in brittle materials toughness is often generated by mechanisms that act at relatively large length scales (micro-scale and up). If we have learned anything from our previous experiences and the observations of Nature it is that the only path to answer these requirements and create new materials is to learn how to build complex architectures on macroscopic dimensions. Here we have shown one example in which a combination of freeze casting with pre-ceramic polymers can be used to form CMG-ceramic composites containing a microscopic network of ‘soft', conductive interfaces. These interfaces replicate two of the aspects observed in nacre, they are very thin (their thickness, few tens of nanometers, is similar to that of the protein layer in nacre) and rough at the submicron level. Cracks propagate through them during fracture and as a result, they generate a series of toughening mechanisms that contribute to a substantial increase in fracture resistance in materials with a minimal carbon content (1 vol%). However, the properties of the soft-layers in nacre and the composites (carbon vs proteins) are quite different. It has been proposed that the soft interfacial phase should exert a ‘lubricant' role to control the sliding of the ceramic bricks^39^. In our materials, carbon can play that role. In multilayer graphene flakes with a similar configuration to the carbon interfaces described in this paper, sliding has been identified as a predominant energy dissipation mechanism^40^. However, the controlled unravelling of the nacre proteins during crack propagation has a contribution to toughness that is different to the one of graphene. In addition, brick design at the microscopic scale is also important as mechanisms such as interlocking have been proposed to contribute to toughness in some nacres^41^. These results underline the need for a systematic, rational comparison of natural materials and their synthetic counterparts to identify the key mechanisms that contribute to the mechanical response and how they interact along the length scales as a way to develop new bioinspired structural materials.

The finely interconnected carbon network provides a highly conductive path that can be used to monitor the materials integrity. It has been shown that graphene networks in relatively soft polymeric matrix materials can provide a path for sensing pressure or bending^42^ and electrical conductivity can also be used to monitor the integrity of polymer-based composites^43^. Here we show how a conductive network can be used to sense damage in a stiff ceramic material. The microscopically connected structure of the network allows sensing of damage at much smaller dimensions (with our conditions a 10 μm crack will give rise to a measurable voltage change of ∼0.1 mV but more sensitivity can be achieved by, for example, increasing the current) and displacements than what has been achieved in polymer-based materials^43^.

A very interesting point raised by our design is that graphene here is not used as a conventional ‘reinforcement' but rather to engineer a fine network of relatively weak interfaces that provide electrical conductivity and fracture resistance. The response depends not only on the chemistry but also on the topology (roughness) of the interfaces at the nano-scale. These results underline the need to look at alternative approaches in the way we design and build practical composites using nanomaterials and that these approaches will need to integrate mechanical and functional response. The latter is probably the most interesting possibility opened by these new layered nanomaterials and a path to generate a step change in the field of structural, multifunctional materials.

## Methods

### Synthesis of large amounts of CMG

Tens of grams of CMG were reproducibly and safely prepared using a modified Tour *et al*.^26^ synthesis in a custom-built rig designed to manipulate up to 10 l of concentrated acids. In a typical synthesis, a 9:1 mixture of concentrated H_2_SO_4_/H_3_PO_4_ (3:0.3 l) was added to 24 g of natural graphite flakes (150–500 μm sieved, Aldrich), followed by the addition of 144 g of KMnO_4_ (6 wt. equiv.). This reaction was slightly exothermic and the temperature rose to 35–40 °C. The reacting suspension was then heated to 50 °C and vigorously stirred at 400 rpm for 18 h. Next, it was cooled to room temperature and the oxidation was stopped by adding dropwise 1.72 l of aqueous H_2_O_2_ (2 wt.%). The graphene oxide suspension was washed using repeated centrifugation at 9,000 rpm (Thermo Scientific Sorvall LYNX 6000 Superspeed Centrifuge) and redispersion in double-distilled water. The work-up was carried out until the supernatant water of the centrifuged CMG was close to pH 6, typically occurring after 16 washing cycles. Low speed (<1,000 r.p.m.) centrifugation cycles were performed to remove any un-exfoliated graphite particles.

### Preparation of rCMG ice templated networks

A suspension of CMG in water (10 mg ml^−1^) was prepared as described above and 0.5 wt.% of Sucrose (S0389 Aldrich) with 0.5 wt.% of poly(vinyl alcohol) (Mw 89,0000-98,000 hydrolysed) with respect to the CMG content were added as binders and structural modifiers. The modified slurry was stirred for 2 h and degassed. It was then poured into a Teflon die and freeze casted at 2 °C min^−1^ to form 33 × 20 × 8 mm^3^ parallelepipeds. The frozen green bodies were freeze dried for 48 h followed by thermal reduction at 900 °C for 1 h in H_2_/Ar atmosphere.

### CMG and freeze casted rCMG characterization

The lateral dimensions of the CMG flakes were measured using optical microscopy and ImageJ software over 100 flakes deposited on a silicon wafer. The CMG content in the slurry was estimated from freeze-dried CMG samples.

### Preparation of rCMG/ceramic composites

The rCMG/ceramic composites were obtained by infiltrating the rCMG networks with a ceramic precursor mixture followed the subsequent polymer-to-ceramic conversion. Infiltration was done in a vacuum caster by immersing the rCMG networks in a solution of polymethylsiloxane (MK Silres—Wacker Chemistry) and a cross-linkable processing aid: methyltriethoxysilane (Sigma Aldrich). Components were mixed in a 2:1 ratio with a small amount (0.1 wt.%) of dibutyltin dilaurate as catalyst. The immersed networks were left to dry in ambient conditions and the polymer formed a gel within 2 days. After gelation, the networks were extracted from the gel and left to dry further in air for 1 week. The drying process was completed by a heat treatment at 200 °C for 1 h in air. Finally, the rCMG/polymer composites were pyrolysed in a tube furnace under flowing nitrogen. Samples were heated to 1,000 °C at 2 °C min^−1^, followed by a 1 h dwell. The obtained rCMG/ Si–O–C composites were left to cool down to room temperature. The samples were further densified using spark plasma sintering (SPS). Composites were placed in standard graphite dies (30 mm in diameter) and embedded in Al_2_O_3_ powder to ensure an even load distribution (Supplementary Fig. 5). The samples were heated to the sintering temperature at a rate of 100 °C min^−1^ with a dwell time of 8 min. The pressure was held constant at 5 MPa to allow degassing. Once the temperature reached 1,400 °C, the pressure was increased to 50 MPa in 3 min.

### Composites characterization

The density of the composites was measured using the Archimedes method. The vol.% of rCMG in the composite was calculated by considering the volume of the rCMG network, density of rCMG and density of the matrix.

X-ray diffraction patterns in the 2*θ* range 10–70° were measured on a Bruker D2 PHASER desktop diffractometer using Cu-Kα radiation, with a total integration time of 1,232 s. Raman spectra were recorded with a WITec confocal Raman microscope using a 532 nm excitation laser source at a laser power of 1 mW. The spectra were collected over an area of 25 × 25 μm^2^ and an average spectrum was calculated.

### Mechanical testing

Rectangular beams were cut from the sintered composites along the freezing direction, using a water cooled diamond blade. The final dimensions of the beams were 25±0.05 mm long (L), 3±0.1 mm wide (W) and 2±0.2 mm thick (B) with the graphene layers parallel to the tension face. All of the beams were polished to a 1 μm finish. Flexural strength was determined by four point bending tests carried out on un-notched beams following the ASTM C1161-13 standard. The tests were conducted at room temperature on a Zwick Roell universal testing machine with a displacement rate of 0.2 mm min^−1^. Both fracture toughness and *R*-curve measurements were measured on single edge V-notched beams (SEVNB). The beams were pre-notched using a water cooled diamond blade and sharpened manually using a razor blade and 1 μm diamond paste.

The specimens for *R*-curve measurement and plane-strain fracture toughness *K*_IC_, where first notched with a diamond blade of 200 μm thickness. The notch was then sharpened using a razor blade using paste of 1 μm. Sharp cracks of almost the half (a/W∼0.5) of the thickness of each specimens were obtained for the *R*-curve measurement to favour a more stable crack propagation^44^. In accordance with the standard (ASTM-E1820−1), the notch sizes of SENB specimen for *K*_IC_ measurement were between 0.12 and 0.3 of W. For the *R*-curve measurements, samples were tested in four point bending (S∼18 mm) with an Instron 8500 universal testing machine at a displacement rate of 0.01 mm min^−1^. The beams were loaded until crack propagation was observed in the load/displacement curve, apparent as a change in the compliance of the material. Afterwards, the specimen was unloaded and the crack was measured with an optical microscope (Zeiss, AxioCam). Different measurements of crack propagation were taken with the precaution of loading the sample always in the same position. The deflections were measured by a linear variable differential transformer.

Nonlinear elastic fracture mechanisms analysis was used to evaluate fracture resistance of the samples as the crack propagated' through the microstructure. The J-integral versus Δ*a* crack extension were estimated. In the J-integral two contributions were taken into account, the elastic J_el_= *K*_IC_^2^/E and the plastic *J*_pl_=1.9A_pl_/Bb where A_pl_ is the plastic area under the load-displacement curve, B the specimen width and b the un-cracked ligament. From the evaluation of *J* it is possible to extract the values of *K* using the *J*–*K* equivalence for nominally mode I fracture (*K*_J_=(*J*·*E*)^1/2^) The same type of evaluation has been used before in other studies such as on bone, nacre-like composite and metallic glasses^45^,^46^.

### Transmission electron microscopy

Work was carried out on a Cs aberration corrected FEI Titan 80–300 S/TEM operated at 300 kV. Transmission electron microscopy (TEM) specimens were prepared from the bulk composite sample by focus ion beam (FIB) milling using a Helios NanoLab 600 instrument (2–30 keV Ga+ incident beam energy with currents of 16 pA–21 nA). To reduce the damage caused by the high energy Ga+ beam and improve the quality of the specimens for subsequent TEM analysis, the specimens were polished at the last stage with 2 keV Ga+ ions.

### X-ray photoelectron spectroscopy

Analyses were performed on CMG and rCMG 3D networks using a K-Alpha spectrometer (ThermoFisher Scientific; East Grinstead, UK). XPS spectra were acquired using a microfocused monochromatic Al Kα X-ray source (hυ=1,486.6 eV). An X-ray spot of ∼400 × 800 μm ellipse shape was used and three different areas were spotted. Core level C1s, C_KLL_, O1s, N1s,Mn1s, S2p, Na1s spectra were acquired using a pass energy of 200 eV and high regions at 40 eV. Casa XPS was used for data interpretation. Shirley or two-point linear background subtractions were employed depending on background shape. Scofield cross-sections were used as relative sensitivity factors in the calculation of the atomic percentages (with RSF of C 1 s=1.000). Peaks were fitted using GL(30) lineshapes; a combination of a Gaussian (70%) and Lorentzian (30%). All XP spectra were charge corrected by referencing the fitted contribution of C-C graphitic like carbon in the C 1 s signal to 284.6 eV. The atomic percentages were calculated from the peak areas in the acquired high resolution C 1 s and O 1 s photoelectron spectra using Scofield sensitivity factors.

### Scanning electron microscopy

The microstructure of the material was analysed with a JSM 6010 LA from JEOL using secondary or backscattered electrons. Green CMG networks were gold coated prior to observations.

### Bulk electrical conductivity

Measurements on the rCMG networks and composites were performed using a four point probe setup composed by a standard bench-top PSU using a constant direct current of 20 mA, and a bench-top multimeter to monitor the voltage drop across the sample.

### Joule heating experiments

Experiments were performed under constant nitrogen flow. Samples were mounted in a custom made setup. As the effective crossectional area of the conductive phase is very low, sufficient pressure and compliant electrodes are used to insure good contact.

### Electrical sensing

Tests were performed on notched beams (25 × 2 × 3 mm^3^) of rCMG/ Si–O–C composite materials by propagating stable cracks on four point bending mode at displacement rate of 0.001 mm min^−1^ allowing for the detection of stable crack growth. The drop of potential across the notch was continuously monitored through the same four point probe apparatus as described above whilst keeping a constant current flow of 20 mA. Crack lengths were measured by pictures taken in the SEM and correlated to the recorded voltage drop during bending.

### Data availability

The data that support the findings of this study are available from the corresponding author upon reasonable request.

## Additional information

**How to cite this article:** Picot, O .T. *et al*. Using graphene networks to build bioinspired self-monitoring ceramics. *Nat. Commun.* **8,** 14425 doi: 10.1038/ncomms14425 (2017).

**Publisher's note:** Springer Nature remains neutral with regard to jurisdictional claims in published maps and institutional affiliations.

## Supplementary Material

Supplementary Information Supplementary Figures

## References

[b1] Coleman J. N., Khan U. & Gun'ko Y. K. Mechanical reinforcement of polymers using carbon nanotubes. Adv. Mater. 18, 689–706 (2006).

[b2] Kuilla T. . Recent advances in graphene based polymer composites. Prog. Polym. Sci. 35, 1350–1375 (2010).

[b3] Porwal H., Grasso S. & Reece M. J. Review of graphene-ceramic matrix composites. Adv. Appl. Ceram. 112, 443–454 (2013).

[b4] Shekhawat A. & Ritchie R. O. Toughness and strength of nanocrystalline graphene. Nat. Commun. 7, 10546 (2016). 26817712 10.1038/ncomms10546 PMC4738364

[b5] Liu Y., Dang Z. H., Wang Y. Y., Huang J. & Li H. Hydroxyapatite/graphene-nanosheet composite coatings deposited by vacuum cold spraying for biomedical applications: Inherited nanostructures and enhanced properties. Carbon 67, 250–259 (2014).

[b6] Mondal J. . Development of a thin ceramic-graphene nanolaminate coating for corrosion protection of stainless steel. Corros. Sci. 105, 161–169 (2016).

[b7] Zhou M. . Highly conductive porous graphene/ceramic composites for heat transfer and thermal energy storage. Adv. Funct. Mater. 23, 2263–2269 (2013).

[b8] Fan Y. C. . Preparation and electrical properties of graphene nanosheet/Al2O3 composites. Carbon 48, 1743–1749 (2010).

[b9] Zhou M. . Heat transport enhancement of thermal energy storage material using graphene/ceramic composites. Carbon 75, 314–321 (2014).

[b10] Zhuang H. . Graphene/3C-SiC Hybrid Nanolaminate. Acs Appl. Mater. Inter. 7, 28508–28517 (2015). 10.1021/acsami.5b09794 26650041

[b11] Centeno A. . Graphene for tough and electroconductive alumina ceramics. J. Eur. Ceram. Soc. 33, 3201–3210 (2013).

[b12] Dusza J. . Microstructure and fracture toughness of Si3N4+graphene platelet composites. J. Eur. Ceram. Soc. 32, 3389–3397 (2012).

[b13] Ramirez C. . Extraordinary toughening enhancement and flexural strength in Si3N4 composites using graphene sheets. J. Eur. Ceram. Soc. 34, 161–169 (2014).

[b14] An Y. . Bioinspired high toughness graphene/ZrB2 hybrid composites with hierarchical architectures spanning several length scales. Carbon 107, 209–216 (2016).

[b15] Belmonte M. . Directional electrical transport in tough multifunctional layered ceramic/grahene composites. Adv. Electron. Mater. 1, 1500132 (2015).

[b16] Wegst U. G. K., Bai H., Saiz E., Tomsia A. P. & Ritchie R. O. Bioinspired structural materials. Nat. Mater. 14, 23–36 (2015). 25344782 10.1038/nmat4089

[b17] Naleway S. E., Porter M. M., McKittrick J. & Meyers M. A. Structural design elements in biological materials: application to bioinspiration. Adv. Mater. 27, 5455–5476 (2015). 26305858 10.1002/adma.201502403

[b18] Evans A. G. Perspective on the development of high-toughness ceramics. J. Am. Ceram. Soc. 73, 187–206 (1990).

[b19] Baskaran S. & Halloran J. W. Fibrous monolithic ceramics.2. flexural strength and fracture-behavior of the silicon-carbide graphite system. J. Am. Ceram. Soc. 76, 2217–2224 (1993).

[b20] Clegg W. J., Kendall K., Alford N. M., Button T. W. & Birchall J. D. A simple way to make tough ceramics. Nature 347, 455–457 (1990).

[b21] Mirkhalaf M., Dastjerdi A. K. & Barthelat F. Overcoming the brittleness of glass through bio-inspiration and micro-architecture. Nat. Commun. 5, 3166 (2014). 24473226 10.1038/ncomms4166

[b22] Bouville F. . Strong, tough and stiff bioinspired ceramics from brittle constituents. Nat. Mater. 13, 508–514 (2014). 24658117 10.1038/nmat3915

[b23] Le Ferrand H., Bouville F., Niebel T. P. & Studart A. R. Magnetically assisted slip casting of bioinspired heterogeneous composites. Nat. Mater. 14, 1172–1179 (2015). 26390326 10.1038/nmat4419

[b24] Barg S. . Mesoscale assembly of chemically modified graphene into complex cellular networks. Nat. Commun. 5, 4328 (2014). 24999766 10.1038/ncomms5328 PMC4102120

[b25] Ni N. . Understanding mechanical response of elastomeric graphene networks. Sci. Rep. 5, 13712 (2015). 26348898 10.1038/srep13712 PMC4562249

[b26] Marcano D. C. . Improved synthesis of graphene oxide. ACS Nano 4, 4806–4814 (2010). 20731455 10.1021/nn1006368

[b27] Ferrari A. C. . Raman spectrum of graphene and graphene layers. Phys. Rev. Lett. 97, 187401 (2006). 17155573 10.1103/PhysRevLett.97.187401

[b28] Bois L., Maquet J., Babonneau F., Mutin H. & Bahloul D. Structural characterization of Sol-Gel derived oxycarbide glasses.1. Study of the pyrolysis process. Chem. Mater. 6, 796–802 (1994).

[b29] Colombo P., Mera G., Riedel R. & Soraru G. D. Polymer-derived ceramics: 40 years of research and innovation in advanced ceramics. J. Am. Ceram. Soc. 93, 1805–1837 (2010).

[b30] Esfehanian M., Oberacker R., Fett T. & Hoffmann M. J. Development of dense filler-free polymer-derived SiOC ceramics by field-assisted sintering. J. Am. Ceram. Soc. 91, 3803–3805 (2008).

[b31] Kleebe H. J., Turquat C. & Soraru G. D. Phase separation in an SiCO class studied by transmission electron microscopy and electron energy-loss spectroscopy. J. Am. Ceram. Soc. 84, 1073–1080 (2001).

[b32] Moysan C., Riedel R., Harshe R., Rouxel T. & Augereau F. Mechanical characterization of a polysiloxane-derived SiOC glass. J. Eur. Ceram. Soc. 27, 397–403 (2007).

[b33] Al Nasiri N. . Effect of microstructure and grain boundary chemistry on slow crack growth in silicon carbide at ambient conditions. J. Eur. Ceram. Soc. 35, 2253–2260 (2015).

[b34] Becher P. F. . Microstructural design of silicon nitride with improved fracture toughness: I, effects of grain shape and size. J. Am. Ceram. Soc. 81, 2821–2830 (1998).

[b35] Cao J. J., MoberlyChan W. J., DeJonghe L. C., Gilbert C. J. & Ritchie R. O. In situ toughened silicon carbide with Al-B-C additions. J. Am. Ceram. Soc. 79, 461–469 (1996).

[b36] Liu H. Y. & Hsu S. M. Fracture behavior of multilayer silicon nitride/boron nitride ceramics. J. Am. Ceram. Soc. 79, 2452–2457 (1996).

[b37] Wilbrink D. V., Utz M., Ritchie R. O. & Begley M. R. Scaling of strength and ductility in bioinspired brick and mortar composites. Appl. Phys. Lett. 97, 193701 (2010).

[b38] Valashani S. M. M. & Barthelat F. A laser-engraved glass duplicating the structure, mechanics and performance of natural nacre. Bioinspir. Biomim. 10, 026005 (2015). 25822595 10.1088/1748-3190/10/2/026005

[b39] Munch E. . Tough, bio-inspired hybrid materials. Science 322, 1516–1520 (2008). 19056979 10.1126/science.1164865

[b40] Rudolph C., Boesl B. & Agarwal A. In situ indentation behavior of bulk multi-layer graphene flakes with respect to orientation. Carbon 94, 872–878 (2015).

[b41] Espinosa H. D. . Tablet-level origin of toughening in abalone shells and translation to synthetic composite materials. Nat. Commun. 2, 173 (2011). 21285951 10.1038/ncomms1172

[b42] D'Elia E., Barg S., Ni N., Rocha V. G. & Saiz E. Self-healing graphene-based composites with sensing capabilities. Adv. Mater. 27, 4788–4794 (2015). 26178801 10.1002/adma.201501653

[b43] Zhang H., Bilotti E. & Peijs T. The use of carbon nanotubes for damage sensing and structural health monitoring in laminated composites: a review. Nanocomposites 1, 167–184 (2015).

[b44] Munz D. & Fett T. Ceramics: Mechanical Properties, Failure Behaviour, Materials Selection Springer (1999).

[b45] Demetriou M. D. . A damage-tolerant glass. Nat. Mater. 10, 123–128 (2011). 21217693 10.1038/nmat2930

[b46] Launey M. E. . Designing highly toughened hybrid composites through nature-inspired hierarchical complexity. Acta Mater. 57, 2919–2932 (2009).

[b47] Porwal H. . Graphene reinforced alumina nano-composites. Carbon 64, 359–369 (2013).

[b48] Wang K., Wang Y. F., Fan Z. J., Yan J. & Wei T. Preparation of graphene nanosheet/alumina composites by spark plasma sintering. Mater. Res. Bull. 46, 315–318 (2011).

[b49] de Andrede M. J. . Carbon nanotube/silica composites obtained by sol-gel and high-pressure techniques. Nanotechnology 19, 265607 (2008). 21828688 10.1088/0957-4484/19/26/265607

[b50] Mukhopadhyay A., Chu B. T. T., Green M. L. H. & Todd R. I. Understanding the mechanical reinforcement of uniformly dispersed multiwalled carbon nanotubes in alumino-borosilicate glass ceramic. Acta Mater. 58, 2685–2697 (2010).

[b51] Ning J. W., Zhang J. J., Pan Y. B. & Guo J. K. Effect of Surfactants on the properties of carbon nanotube-reinforced SiO2 matrix composites. Key Eng. Mater. 249, 61–64 (2003).

[b52] Zhan G. D., Kuntz J. D., Wan J. L. & Mukherjee A. K. Single-wall carbon nanotubes as attractive toughening agents in alumina-based nanocomposites. Nat. Mater. 2, 38–42 (2003). 12652671 10.1038/nmat793

[b53] Belmonte M. . Toughened and strengthened silicon carbide ceramics by adding graphene-based fillers. Scripta Mater. 113, 127–130 (2016).

[b54] Walker L. S., Marotto V. R., Rafiee M. A., Koratkar N. & Corral E. L. Toughening in graphene ceramic composites. ACS Nano 5, 3182–3190 (2011). 21443241 10.1021/nn200319d

[b55] Porwal H. . Tribological properties of silica-graphene nano-platelet composites. Ceram. Int. 40, 12067–12074 (2014).

[b56] Guo S. Q., Sivakumar R., Kitazawa H. & Kagawa Y. Electrical properties of silica-based nanocomposites with multiwall carbon nanotubes. J. Am. Ceram. Soc. 90, 1667–1670 (2007).

[b57] Inam F., Yan H., Reece M. J. & Peijs T. Dimethylformamide: an effective dispersant for making ceramic-carbon nanotube composites. Nanotechnology 19, 195710 (2008). 21825728 10.1088/0957-4484/19/19/195710

[b58] Miranzo P. . In situ processing of electrically conducting graphene/SiC nanocomposites. J. Eur. Ceram. Soc. 33, 1665–1674 (2013).

[b59] Ramirez C., Figueiredo F. M., Miranzo P., Poza P. & Osendi M. I. Graphene nanoplatelet/silicon nitride composites with high electrical conductivity. Carbon 50, 3607–3615 (2012).

[b60] Thostenson E. T., Karandikar P. G. & Chou T. W. Fabrication and characterization of reaction bonded silicon carbide/carbon nanotube composites. J. Phys. D Appl. Phys. 38, 3962–3965 (2005).

[b61] Zhan G. D., Kuntz J. D., Garay J. E. & Mukherjee A. K. Electrical properties of nanoceramics reinforced with ropes of single-walled carbon nanotubes. Appl. Phys. Lett. 83, 1228–1230 (2003).

[b62] Tatami J., Katashima T., Komeya K., Megure T. & Wakihara T. Electrically Conductive CNT-Dispersed Silicon Nitride Ceramics. J Amer Ceram. Soc 88, 2889–2893 (2005).

