# Supplementary material for: Using graphene networks to build bioinspired self-monitoring ceramics
Source: Nat Commun. 2017 Feb 9;8:14425. doi: 10.1038/ncomms14425 (PMC5309856; doi:10.1038/ncomms14425)
Supplement: Supplementary Information — Supplementary Figures [file ncomms14425-s1.pdf]

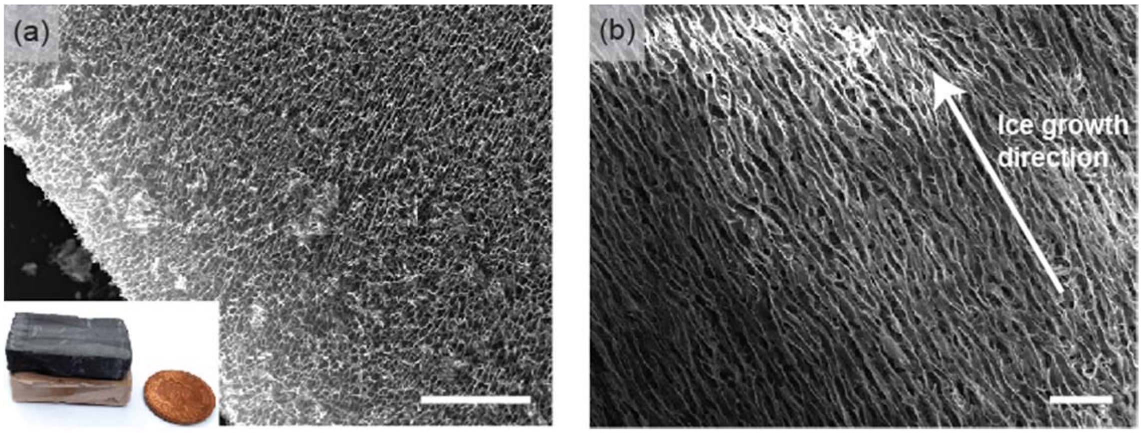

Supplementary Figure 1. Low magnification images of the graphene network in the directions (a) perpendicular and (b) parallel to the ice growth. The insert in (a) shows pictures of the foams before (bottom) and after (top) thermal reduction. Scale bars, 500 µm **(a)**, 100 µm **(b)**.

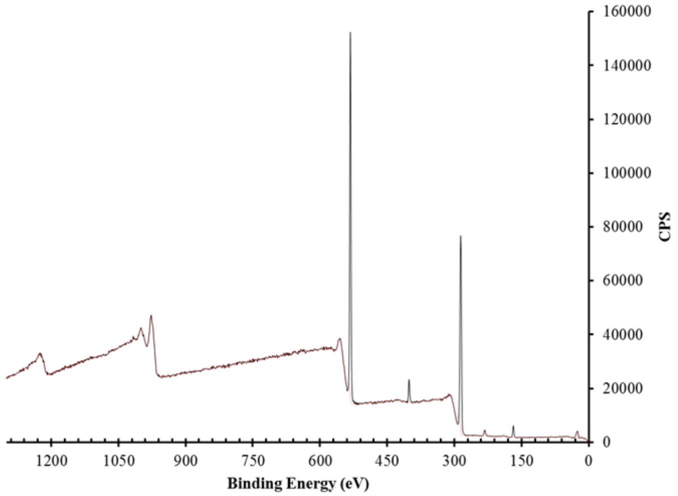

Supplementary Figure 2. XPS survey of freeze dried CMG. Atomic composition C1s (53.5%), N1s (3.5%), O1s(38.8%), S2p (4.2%). Sulphur and Nitrogen are attributed to the vacuum grease used during the freeze casting process while the small fraction of Sulphur can be a residue from the synthesis route.

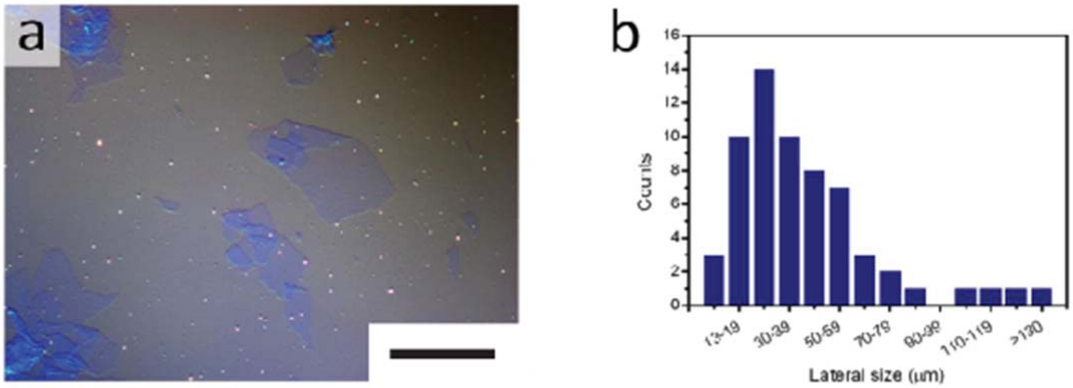

Supplementary Figure 3. CMG flake size analysis. (a) Optical microscope image of CMG flakes on Si/SiO_2 substrate obtained by differential interference contrast. (b) Lateral size distribution for 100 CMG flakes. Scale bar, 50 µm **(a)**.

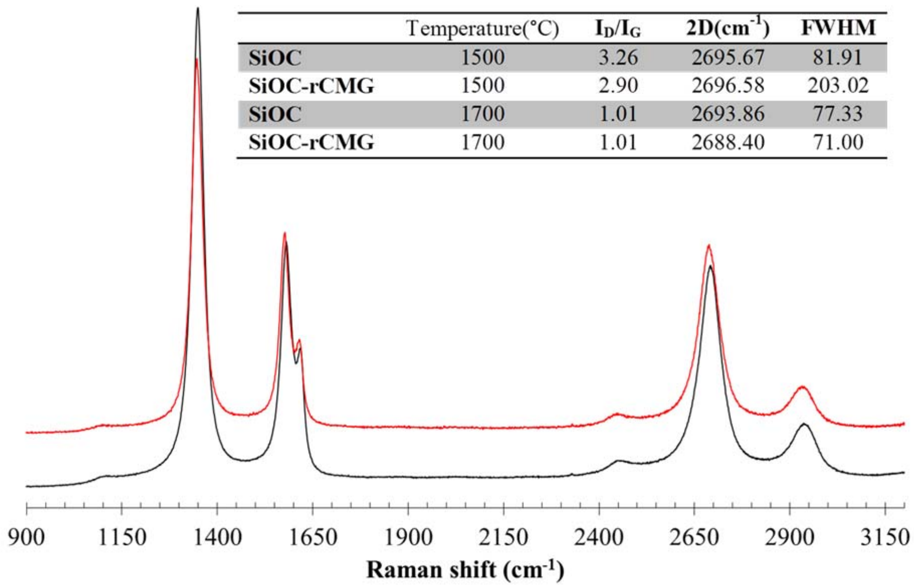

	Temperature(°C)	I_D/I_G	2D(cm^{-1})	FWHM
**SiOC**	1500	3.26	2695.67	81.91
**SiOC-rCMG**	1500	2.90	2696.58	203.02
**SiOC**	1700	1.01	2693.86	77.33
**SiOC-rCMG**	1700	1.01	2688.40	71.00

Supplementary Figure 4. Raman study of SiOC-rCMG composites. Raman spectra of pure matrix SiOC (black) and composite (red) at 1700 °C. Inset table. Calculated Raman parameters at 1500 °C and 1700 °C.

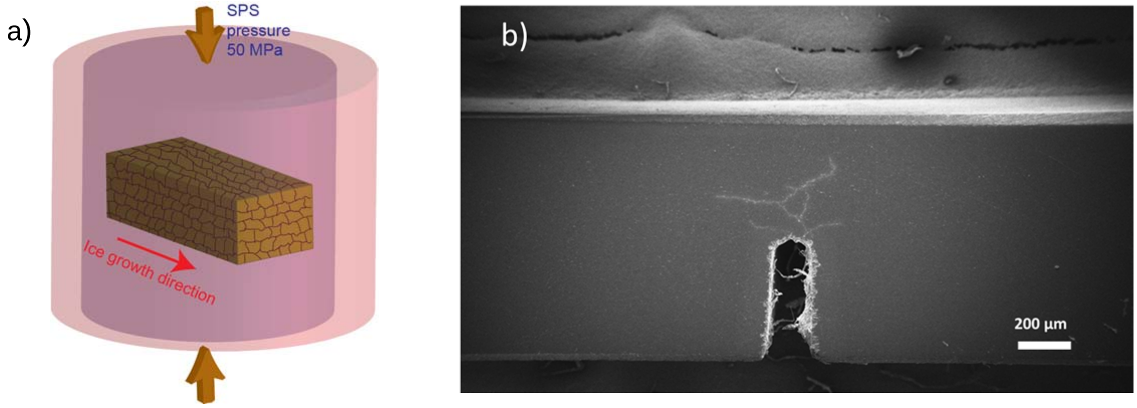

Supplementary Figure 5. Spark Plasma Sintering layout and SENB dead-specimen micrograph. (a) Schematic illustration of the infused foams into the SPS graphite die and (b) notched sample micrograph after R-curve testing
